# Echoes of the Month

**Published:** 1923-08

**Authors:** 


### ECHOES OF THE MONTH.

### PUBLIC HEALTH OF MANCHESTER.

Under the title of "Observations in the History of Public Health Effort in Manchester," Dr. James Niven, who was medical officer of health for the city from 1894 until last year, when he retired, has compiled a report which deals at length with many phases of his subject. The removal of organic filth has had, he thinks, most to do with the improvement of the public health and the control of infectious diseases. Objects still to be attained include an adequate disinfecting station and a smallpox hospital, which should be so situated as to be well separated from dwellings, and should be so constructed and situated as to serve also as a tuberculosis hospital. Increased activity is needed in removing sepsis. For that reason the tipping of organic undestructed matter within the city should be abandoned. Increased effort should be bestowed on places engaged in the making of foods and drinks, on restaurants, cafés, etc., with a view to protect the public from contaminated foods. The investigation of pneumonia from the clinical and public health sides requires attention, but should only be attempted with the aid of a highly-qualified medical investigator.

### MEDICAL TREATMENT FOR SECONDARY SCHOOL PUPILS.

The School Medical Officer for Glamorgan (Dr. E. Colston Williams), in his annual report for last year, states that the secondary school pupils of the county show a considerable amount of defective vision and dental disease. Examination was made of 6,121 pupils, and the number requiring treatment, or to be kept under observation, was as high as 2,871. Of these 344 were found to be suffering from defects of vision, 169 from defects of the nose and throat, and 1,994 from dental defects. Dr. Williams also reports a considerable amount of anæmia and of spinal curvature, especially among the girls—the number of cases of anæmia being sixty-three and of spinal curvature ninety-two. It would have been interesting to have a report upon the causes of these defects in the secondary schools and of their relationship, if any, to the school work. The Glamorgan authority has not yet made any arrangements for the treatment of these cases, though during the year permission was granted to secondary pupils to receive treatment at the clinics on the same terms as those provided for elementary school children. It is becoming increasingly clear that treatment of the secondary pupils is as necessary as it is for the elementary, and that the mere inspection of the pupils, unless followed by effective preventive measures, is almost valueless.

### EXIT THE FAINTING LADY.

What has become of the early Victorian fainting-fit ? asks the Dean of St. Paul's. Did our grandmothers really faint, or was it only a well-worked means of attraction for the other sex ? The fainting-fit seems to have succeeded "the spleen" and "the vapours," which can hardly have been very engaging. But fits of screaming seem to have attracted not only the attention but the compassion of our gallant ancestors, who regarded woman as a cross between an angel and an idiot, and petted her when she gave way to the weaknesses which were supposed to belong to her sex. Will our grandchildren look back upon our ideas about health as we look upon the early Victorians ? I think not. Perhaps the present fad for removing superfluous organs may go out of fashion ; but modern hygiene is based on solid knowledge. There are just 189 recognised ways of dying ; a doctor who lets a patient die in any other way has to explain himself to the Registrar-General.

### A NORWEGIAN RED CROSS HOSPITAL SHIP.

A discarded Norwegian battleship, *The Viking*, has been transformed into a hospital ship and it has now begun its work of mercy among the fishermen in the north of Norway, where the Norwegian Red Cross cannot afford to establish mobile hospitals, but where there is a great need of them. *The Viking* has beds for 50 patients and is in every respect well equipped ; it has a big modern operating theatre, dispensary, baths, delousing room, disinfection room, laundry, convalescent ward and all other necessary fittings. Two of the wards are exceedingly comfortable, entirely furnished as they are with material from mobile hospitals of the King and Queen of Norway. The Norwegian Red Cross hopes that *The Viking* may, in the first place, improve public health in northern Norway and lessen disease among the fishermen there. But it hopes also that it will spread the idea of the Red Cross successfully.

### LEPROSY IN INDIA.

Writing in the Annual Report of the Mission to Lepers, Dr. Muir (Leprosy Research Worker in the School of Tropical Medicine, Calcutta) says :—Within the last few months I have come across cooks and house-servants, schoolboys attending school, students attending college, teachers of both sexes instructing the young, confectioners serving over the counter and men and women in other walks of life all in a position to spread the disease to those around them. In a well-known institution in Calcutta, out of a menial staff of sixty I found four suffering from leprosy. One boy acquired the disease by playing about on matting left in a house by the previous tenant who was found on inquiry to have been a well-advanced leper. A child of three now attending our dispensary has a small leprous patch on the cheek acquired by lying on the bed of an uncle suffering from the disease. Illustrations could be multiplied, but the important point is that a searchlight is being turned on to the disease as never before, and ignorance and shame will not be able to hold up their heads. It would be a great pity to use unwise compulsion in the segregation of lepers, but it is the duty of the State and of the public to provide retreats for those lepers who wish to save their friends and the public from the danger of contagion and have not the means of isolating themselves in their own homes.

### MINNOWS TO FIGHT MOSQUITOES.

The use of fish to destroy the larvæ of the mosquito in waters where they breed has been often advised and employed to some extent. A minnow especially adapted to this use, being vigorous, prolific and very hungry for mosquitoes, is now bred on a large scale in California, and it is hoped that its use may relieve mosquito-ridden communities where for any reason crude oil or other means of fighting the pest are not available. It feeds largely upon insects, and wherever it inhabits water in which mosquitoes breed, its principal food consists of mosquito larvæ. For the destruction of mosquito larvæ it has been found superior to any other species. Because of its extreme prolification, easy propagation, ability to adapt itself to different conditions and to reach areas not penetrated by other species, exceptional devouring capacity, general habits and living in identical areas with mosquito larvæ, "Gambusia affinis" is the most valuable natural agent known for the abatement of mosquito-breeding.

### POSTURE IN WOMEN'S WORK

The effect on the physique and on the health of industrial workers of the monotonous and rapid repetition hour after hour and month after month of movements in themselves light and simple has recently begun to challenge the attention of the medical profession. Especially has it done this since the work, so characteristic of modern industry, has been more and more monopolised, unusually on a piece basis, by women whose deftness and swiftness have enabled them to take possession of occupations which did not require much muscular strength. So far, although conclusive proof is lacking, the weight of opinion seems to be that serious injury may be caused by any work, the performance of which requires cramped, constrained or awkward posture. Bad results may be prevented or lessened by change of work ; for instance, a change from work that requires stooping forward to work that requires reaching upward, or that requires a sideways slouch, or that requires no particular attitude. Proper chairs are also helpful. All chairs, for instance, that are too high to permit the occupant's feet to rest firmly on the floor should be provided with a foot rest that would prevent the lower part of the legs from dangling and the thighs from pressing against the edge of the chair ; practically this means that women should not be called upon to use chairs built for men. That the constant repetition of a movement does in time affect the nervous system is more than probable, but how importantly it affects it has not been established. It is stated that girl after girl in a cigarette packing factory was found when off duty to be constantly repeating the motions she made while at work, and such tendencies should be investigated.

### HYGIENE IN FRENCH SCHOOLS.

The recent Congress of Hygiene in Paris expressed the most admirable sentiments, but, as a Frenchwoman recently remarked, in actual practice the State schools and lycées are most of them in a deplorable condition as regards washing and sanitary accommodation for the children. There is rarely a nurse in any of them; where washing accommodation is supplied there is no soap; if a child is hurt there is nobody but an untrained maid to look after him. In the newer schools elaborate washing arrangements are made for the children. Sometimes they are so elaborate, so model, that they do not seem to belong to the real life of mere soap and water. In the various crèches, rest-rooms, and in the big new institutions, hygiene is made a point of, but in so pedantic a fashion that it is too boring to children for them to take to it very readily.

### FAT MEN BORN, NOT MADE.

Fat men are born, not made, according to Dr. C. B. Davenport, director of the Station for Experimental Evolution at Cold Spring Harbour, New York. According to his statement, the only safe and sure way to be always slim and sylphlike in figure is to have parents who were slender in build. But fat persons may have slender children, or rather, children who will always remain slender. On the other hand, children of two thin persons will never grow fat. Dr. Davenport's conclusions were questioned to some extent by Prof. Graham Lusk of the Cornell University Medical School, who asserted that appetite and nutrition had much to do with putting on weight. "If a man drinks one-third of a glass of milk above his nutritional requirements every day, he will gain 9 pounds in a year and 90 pounds in 10 years," he said. "Appetite has much to do with it. We have hot summers in this country; a man has little appetite during them, and a fat man in hot weather cannot do his work as well as a thin one. That is why the typical American, 'Uncle Sam,' is thin, while the Britisher, typified by 'John Bull,' is portly."

### WHY BREAD GROWS STALE.

Old ideas of how bread grows stale are overturned by the Food Research Institute in its first publication just issued from its headquarters at Stanford University, U.S.A. The report points out the wholesomeness of the stale loaf, shows the waste produced by present bakery practices, and urges further investigation of why some bread keeps better than others. The Science Service's "Daily Science News Bulletin" says:—"What probably occurs is that much of the moisture in the bread is held by the starch which has been gelatinized in the baking. As the loaf comes out of the stove, this starch jelly distributed through the bread contains all the moisture it can hold. As the bread cools, the starch gives up some of its moisture and this moisture is absorbed by the other constituents of the loaf, changing the crust from a brittle material that crunches between the teeth to a soft and pliable one, while the gluten of the crumb is given a toughness and firmness which as fresh bread it did not have. The bread becomes stale at low temperatures, and this accounts for the fact that bread when stale, but not dry, can be freshened up by heating. The process is reversed and the starch-jelly reabsorbs the moisture from the other bread constituents."

### CULTIVATING THE ANGELS.

When distributing prizes and certificates awarded by the *Yorkshire Evening Post* to the hundred best babies of the Leeds Babies' Day Competition, Sir Berkeley Moynihan said that babies come straight from heaven and were the nearest approach to angels that this imperfect world could show. It was necessary that these angels should be cultivated when they arrived, and the future would show that baby culture was really the test of the progress of civilisation in any country. Bismarck had once said that the mind of a child was like a white sheet of paper, and one could write on it, in indelible characters, exactly the message wished. What was true of a child's mind was equally true of a child's body: the body recorded the history of its ancestors, and especially of its nearest ancestors. The greatest thing that could be done for a child's body was done by the exercise of the finest craftsmanship that the world could show. It was a very fine thing to be a craftsman in any art. It was the best thing of all to work in the finest material in the world and mould it at one's own will, as a surgeon did; but, after all, the greatest craft was mothercraft.

### HEALTH OF EUROPEAN CHILDREN IN KENYA.

Dr. Mackinnon, until recently of the East African Medical Service, has been carrying out an investigation on education in its relation to the physical and mental development of European children of school age in Kenya, studying for the purpose 500 children born and bred in the Highlands of the Colony. He found that their general physique is, on the average, much above that of children in England. There is, unfortunately, a different story to tell when the effects on the nervous system are concerned. At first, indeed up to the age of 10, all is well. There is even a precocious intellectual development. Thereafter, however, a gradual process of mental exhaustion and deterioration supervenes, which appears especially to affect memory and the power of application. Accordingly, he recommends that, save in the case of children whose circumstances are such that they cannot be sent home, secondary education, like that carried out in this country, should not be encouraged.

### HOSPITALS AND TEXTILE SUPPLIES.

The hospital world has become a vast purchaser of drapery supplies. In the United Kingdom there are over 270,000 beds for which equipment is needed, and at least another 30,000 for the staff. The initial stocking of these represents, the *Drapers' Record* calculates, over a million and a-half pounds. On the top of this there comes the annual expenditure on dressings and bandages, consisting of wool, lint and gauze tissue, which reaches the value of £1,410,000. Beyond this, there is a provision made in poor law infirmaries and infectious and mental hospitals for nightdresses and underlinen and print dresses for the inmates. This covers an expenditure of £100,000, and possibly £150,000. Then there is a necessary provision for brushes and brooms, dusters and teacloths and scrubbers, which must run into a very large amount. It is not possible to estimate the huge expenditure on material for blinds, or the large contribution that must be made by purchases of waterproofed cushions and beds, and miscellaneous expenditures such as screens. The cost of nurses' uniform, too, works out at over £700,000 per annum.

### WOMAN PHYSICIAN FOR COASTGUARD DUTY.

Dr. Blanche N. Epler, residing at Hatteras, North Carolina, has been appointed by the United States Public Health Service as contract physician to furnish professional services to several coastguard stations. Dr. Epler is engaged in private practice among the inhabitants of this somewhat isolated and exposed region. She will be prepared to respond at any time, day or night, to calls arising out of any serious accidents happening to coastguardsmen in the course of their arduous tasks. She will also conduct the visual and other physical examinations of applicants for admission to the coastguard service at the stations under her medical supervision. Dr. Epler was recommended for the duty by the local District Superintendent of the coastguard.

### A FRESH-AIR COLONY FOR CHILDREN.

The St. Chamend committee, in the Department of the Loire, which has already organised a very efficient anti-tuberculosis centre, including a dispensary, a sanatorium for women and a small open-air hospital for children slightly infected with tuberculosis, has, with the help of local business men, established a mountain fresh-air colony. This establishment is situated at an altitude of 3,600 feet above sea level, on a vast wooded plateau, in a district well known for its excellent climate since the "Children's Mountain Home" was so successfully established there. It is open only during the summer months and acts as a preventorium by giving a very effective fresh-air cure to 700 children sent by the St. Chamend dispensary.

